# Factors associated with blood lead levels in children in Shenyang, China: a cross-sectional study

**DOI:** 10.1186/s12887-022-03182-9

**Published:** 2022-03-10

**Authors:** Xiao-Jun Cheng, Guang-Bo Li, Shuang-Shuang Zhang, Ying Liu, Yi-Chen Dong, Xue-Ning-Li

**Affiliations:** 1 Department of Pediatrics, The Fourth Affiliated Hospital of China Medical University, Shenyang, Liaoning People’s Republic of China; 2 Department of Child and Adolescent Health, School of Public Health, China Medical University, Shenyang, Liaoning People’s Republic of China

**Keywords:** Children, Blood lead levels, Diet

## Abstract

**Background:**

Although blood lead levels (BLLs) in children are gradually decreasing, low-concentration lead exposure can still exert adverse effects. We studied the factors that affect BLLs in children in Shenyang, China.

**Methods:**

We conducted a cross-sectional study by administering structured questionnaires on family demographics and food intake. The concentrations of lead in venous blood were determined by graphite furnace atomic absorption spectrometry.

**Results:**

A total of 273 children aged 1–6 years were enrolled. The geometric mean (geometric standard deviation) of BLLs was 24.94 (12.70) μg/L in boys and 23.75 (11.34) μg/L in girls. The prevalence of BLLs of ≥35 μg/L was 22.7% and was mainly observed in children aged under 3 years. Often hand washing before meals was protective against BLLs ≥20 μg/L (adjusted OR: 0.427, 95%CI: 0.238–0.767, *p* = 0.004). Consumption of puffed grains and eggs had an adjusted OR (95%CI) for BLLs ≥20 μg/L of 1.714 (1.012–2.901) (*p* = 0.045) and 1.787 (1.000–3.192) (*p* = 0.050), respectively.

**Conclusions:**

BLLs of children in Shenyang are still higher than in developed countries. Consumption of puffed grains and eggs is associated with higher BLLs. Often hand washing before meals may be protective against high BLLs.

## Background

Lead is considered one of the most toxic substances by the US Agency for Toxic Substances and Disease Registry [1]. The Chinese government began to focus on lead poisoning among children in the 1990s and blood lead levels (BLLs) in children have declined significantly since the introduction of unleaded gasoline [2]. BLLs are declining globally with the implementation of policies to reduce the risk of lead poisoning, and Centers for Disease Control and Prevention has recently revised the BLLs reference value from 50 to 35 μg/L based on the latest National Health and Nutrition Examination Survey data [3]. As lead exposure levels have declined, research into lead sources has tapered off. However, low-concentration lead exposure can also exert adverse effects on children, such as lower intelligence quotient and symptoms associated with attention-deficit/hyperactivity disorder [4, 5], and increase the risk of respiratory infections in early life [6]. Overall, there is still a need to elucidate the factors affecting BLLs and reduce exposure levels. Food is currently considered a major source of lead, as environmental sources have gradually declined [7]. To our knowledge, few reports have investigated the factors that influence BLLs in children, especially dietary factors [8]. In the present study, we assessed sociodemographic and dietary factors that may influence BLLs in children in Shenyang, China.

## Methods

This was a cross-sectional study conducted in children aged 1–6 years at the physical examination center of the Fourth Affiliated Hospital of China Medical University. The criteria for inclusion were children who were found to be healthy by medical examination and whose BLLs were measured. Children were excluded if they had received zinc, calcium, iron, or multivitamin supplementation during the preceding 3 months. The study period was December 2017 to December 2019. A total of 305 children were approached while 32 had the exclusion criteria; 273 children were eventually enrolled in the study. Ethics approval was granted by the Ethics Committee of the Fourth Affiliated Hospital of China Medical University and written informed consent was obtained from the parents/caretakers of the participants.

A total sample size of 273 children was determined with a first error level of 5%, a standard deviation of 15 and accuracy of 12% of standard deviation error.

The parents/caretakers of the participants completed a questionnaire, including information on the child’s age, sex, one-child family (yes/no), passive exposure to cigarette smoke (one or more family members had regularly smoked at home), socioeconomic status (low/above median income), maternal education (≤ high school/college or higher), parents’ occupations (unemployed, factory workers, service workers including social and catering service workers, or intellectual), whether the home had been remodeled within the past year, and frequency of hand washing before meals (“often” means washing hands at least twice a day, “seldom or not” means washing hands less than 2 times a day). Body mass index (kg/m^2^) was calculated by the hospital staff from height and weight measurements. Age- and sex-specific body mass index cutoffs developed for Chinese children were used to evaluated participant weight status (normal, overweight and obese) [9].

A food frequency questionnaire was used in the present study. The original version of the food frequency questionnaire which is both reproducible and valid included 86 food items that were commonly consumed by the Chinese population [10, 11], while 17 food items were selected into this study to assess the average consumption frequency over the past month. The food categories included rice/wheat, potatoes, vegetables, fruit, poultry, seafood, dairy products, eggs, bean products, nuts, whole grains, animal liver, puffed grains, pickles, fried food, carbonated beverages, and desserts. Intake frequency choices were never, one to three times per month, one to two times per week, three to five times per week, and at least once a day.

### Laboratory measures

Venous blood (4 mL) was collected by a phlebotomy nurse to determine the concentration of blood lead. Blood was collected into lithium heparin-coated trace metal-free tubes (Shenyang Baokang Biological Engineering Co., Ltd., China) and transported on ice to Shenyang Harmony Health Medical Laboratory. The testing credentials of the laboratory have been previously described [12]. BLLs were determined by atomic absorption spectrometry through graphite furnace ionization (Beijing Bohui Innovation Biotechnology Co., Ltd., China), relying on the available volume of whole blood. BLLs higher than the reference value of 50 μg/L were re-measured to rule out possible contamination during specimen processing.

### Statistical analysis

**Fig. 1:**
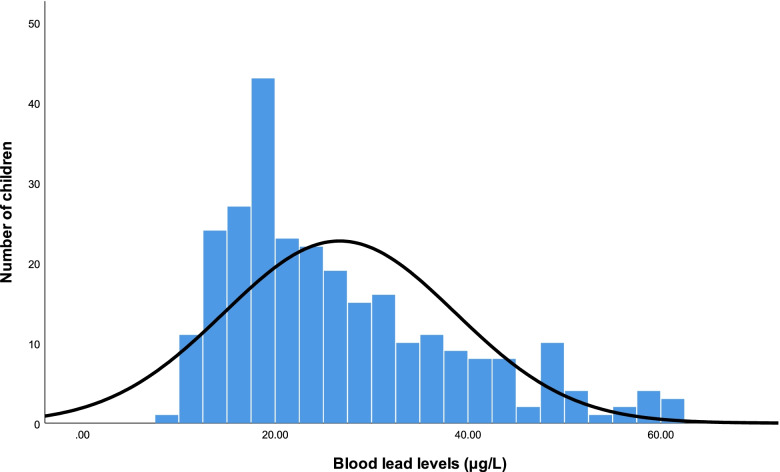
Distribution of blood lead levels in study participants

BLLs were not normally distributed (Fig. 1), and therefore BLL data were quantified by geometric mean ± geometric standard deviation and compared by *t*-test after logarithmic transformation. The chi-squared test was used to compare qualitative data and descriptive statistics are shown as n (%). Gilbert and Weiss have proposed 20 μg/L as a benchmark for successful prevention, therefore we took dichotomous BLLs as the dependent variable (< 20 μg/L and ≥ 20 μg/L) [13]. Food intake was dichotomized by the average intake frequency of each item. A multivariate logistic regression model was constructed and covariate selection showed a univariate relationship with BLLs (*p* ≤ 0.2). Maternal education, hand washing frequency, and consumption of fruit, bean products, puffed grains, and eggs were included in the multivariate logistic regression model. Statistical analyses were performed in SPSS version 22.0 (IBM SPSS, Armonk, NY, USA). *p* ≤ 0.05 was considered significant.

## Results

A total of 273 children participated in this study, including 126 boys (46.2%) and 147 girls (53.8%). The participants ranged in age from 1 to 6 years (mean: 4.00 ± 1.13 years); 108 children (39.6%) were aged 1–3 years and 165 children (60.4%) were aged 4–6 years. There was no statistical difference in the distribution of boys and girls between the two age groups (χ^2^ = 3.262, *p* = 0.660).

Figure 1 shows the distribution of BLLs. The geometric mean (geometric standard deviation) of BLLs was 24.94 ± 12.70 μg/L in boys and 23.75 ± 11.34 μg/L in girls. No statistical difference in BLLs was found between sexes in either age group (Table 1). The BLLs of 62 samples (22.7%) were greater than or equal to 35 μg/L. The prevalence of BLL ≥35 μg/L was higher in the 1–3 years group (34 samples, 31.5%) than in the 4–6 years group (28 samples, 17.0%), and the difference was statistically significant (χ^2^ = 7.831, *p* = 0.005). However, no significant difference in BLLs was observed between the two age groups (*p* > 0.05; Table 1).

**Table 1:** Blood lead levels of study participants

Age (year, N)	Blood lead levels (GM ± GSD, range)		Total (GM ± GSD, range)	*p#*
	Boy	Girl		
1–3 (108)	25.64 ± 14.86(10.41–60.43)	24.85 ± 12.98(10.80–61.85)	25.17 ± 13.73(10.41–61.85)	0.737
4–6 (165)	24.58 ± 11.37(10.55–61.57)	22.94 ± 9.82(8.98–49.50)	23.74 ± 10.63(8.98–61.57)	0.271
Total (273)	24.94 ± 12.70(10.41–61.57)	23.75 ± 11.34(8.98–61.85)	24.30 ± 11.99(8.98–61.85)	0.351
p*	0.609	0.256	0.274	

*Independent samples *t*-test for blood lead levels between age groups. #Independent samples *t*-test for blood lead levels between sexes. *GM* geometric mean, *GSD* geometric standard deviation

In the crude model, often hand washing before meals was more likely to be associated with BLLs below 20 μg/L than lower frequencies (crude odds ratios (OR): 0.382, 95% confidence intervals (CI): 0.218–0.671, *p* = 0.001) (Table 2). Consumption of puffed grains (at least once per week) had a crude OR for BLLs ≥20 μg/L of 1.816 (95%CI: 1.102–2.992) (*p* = 0.019) (Table 3). In the multivariate logistic regression model, the association between often hand washing before meals or consumption of puffed grains (at least once per week) and BLLs ≥20 μg/L remained (adjusted OR for often hand washing before meals: 0.427, 95%CI: 0.238–0.767, *p* = 0.004; adjusted OR for consuming puffed grains: 1.714, 95%CI: 1.012–2.901, *p* = 0.045) (Table 4). Moreover, the association between consumption of eggs (≥1 times/day) and BLLs of ≥20 μg/L was borderline significant in the crude model (Table 3), but when adjusted for confounders, the association became significant (adjusted OR: 1.787 (95% CI: 1.000–3.192, *p* = 0.050) (Table 4).

**Table 2:** Univariate logistic regression model of probability of blood lead levels ≥20 μg/L

Variable		*N*	%	Crude OR	95%CI	*p*-value
Sex	boy	126	46.2	1		
	girl	147	53.8	0.762	0.467–1.243	0.276
Age (years)	1–3	108	39.6	1		
	4–6	165	60.4	1.348	0.822–2.213	0.237
Body mass index	normal	211	77.3	1		
	overweight and obese	62	22.7	0.863	0.485–1.534	0.615
One-child Family	yes	225	82.4	1		
	no	48	17.6	1.091	0.574–2.074	0.791
Passive smoking	yes	174	63.7	1		
	no	99	36.3	0.758	0.458–1.253	0.280
Social economic status	low	61	22.3	1		
	above median income	212	77.7	1.008	0.563–1.806	0.978
Maternal education	≤high school	70	25.6	1		
	college or higher	203	74.4	0.636	0.357–1.132	0.124
Maternal occupation	unemployed	59	21.6	1		
	factory workers	91	33.3	0.760	0.381–1.516	0.436
	service workers	69	25.3	0.618	0.299–1.274	0.192
	intellectual	54	19.8	0.640	0.297–1.379	0.640
Paternal occupation	unemployed	15	5.5	1		
	factory workers	130	47.6	1.067	0.358–3.178	0.908
	service workers	94	34.4	1.074	0.353–3.271	0.900
	intellectual	34	12.5	0.844	0.246–2.904	0.788
Newly decorated house	yes	22	8.1	1		
	no	251	91.9	1.323	0.551–3.180	0.532
Hand washing before meals	seldom or not	89	32.6	1		
	often	184	67.4	0.382	0.218–0.671	0.001

*CI* confidence interval, *OR* odds ratio

**Table 3:** Relationship between food intake and blood lead levels ≥20 μg/L

Variable		N	%	Crude OR	95%CI	*p*-value
Rice/wheat	<1 times/day	30	11.0	1		
	≥1 times/day	243	89.0	1.039	0.479–2.253	0.924
Potato	≤2 times/week	187	68.5	1		
	>2 times/week	86	31.5	0.792	0.471–1.332	0.380
Vegetables	<1 times/day	103	37.7	1		
	≥1 times/day	170	62.3	0.856	0.517–1.416	0.545
Fruit	<1 times/day	56	20.5	1		
	≥1 times/day	217	79.5	0.615	0.327–1.155	0.131
Poultry	≤2 times/week	98	35.9	1		
	>2 times/week	175	64.1	0.973	0.586–1.615	0.916
Seafood	≤2 times/week	222	81.3	1		
	>2 times/week	51	18.7	0.818	0.441–1.515	0.523
Dairy	<1 times/day	109	39.9	1		
	≥1 times/day	164	60.1	0.895	0.544–1.472	0.663
Bean products	≤2 times/week	194	71.1	1		
	>2 times/week	79	28.9	1.581	0.910–2.750	0.104
Eggs	<1 times/day	178	65.2	1		
	≥1 times/day	95	34.8	1.610	0.953–2.722	0.075
Nuts	≤2 times/week	183	67.0	1		
	>2 times/week	90	33.0	1.259	0.746–2.123	0.388
Whole grains	<1 times/week	97	35.5	1		
	≥1 times/week	176	64.5	0.999	0.601–1.659	0.996
Animal liver	never	97	35.5	1		
	≥1 times/month	176	64.5	1.304	0.787–2.161	0.303
Puffed food	<1 times/week	152	55.7	1		
	≥1 times/week	121	44.3	1.816	1.102–2.992	0.019
Pickles	never	139	50.9	1		
	≥1 times/month	134	49.1	0.807	0.496–1.313	0.388
Fried food	<1 times/week	212	77.7	1		
	≥1 times/week	61	22.3	0.832	0.467–1.484	0.534
Carbonate beverages	never	137	50.2	1		
	≥1 times/month	136	49.8	0.901	0.554–1.465	0.674
Dessert	<1 times/week	108	39.6	1		
	≥1 times/week	165	60.4	0.806	0.489–1.330	0.399

*CI* confidence interval, *OR* odds ratio

**Table 4:** Multivariate logistic regression model of probability of blood lead levels ≥20 μg/L

Variable		Adjusted OR^a^	95%CI	*p*-value
Maternal education	≤high school	1		
	college or higher	0.650	0.355–1.188	0.161
Hand washing before meals	seldom or not	1		
	often	0.427	0.238–0.767	0.004
Fruit	<1 times/day	1		
	≥1 times/day	0.622	0.317–1.221	0.168
Bean products	≤2 times/week	1		
	>2 times/week	1.449	0.797–2.632	0.224
Puffed food	<1 times/week	1		
	≥1 times/week	1.714	1.012–2.901	0.045
Eggs	<1 times/day	1		
	≥1 times/day	1.787	1.000–3.192	0.050

^a^Adjusted for maternal education, hand washing frequency, and consumption of fruit, bean products, puffed grains, and eggs. *CI* confidence interval, *OR* odds ratio

## Discussion

Lead is a ubiquitous metal and its blood concentrations are considered the best indicator of human exposure. No BLLs are considered safe in children, who are particularly susceptible to lead poisoning [14, 15] because their organ systems are still developing. Lead exposure is often unrecognized because it typically occurs without obvious symptoms. BLLs in children aged ≤10 years ranged from 10.98 to 511.2 μg/L (mean: 135.59 μg/L) in Shenyang in the year 2000 [16]. In the present study, we found lower BLLs, perhaps as a result of the implementation of unleaded gasoline policies and relocation of heavy industries. However, child BLLs in China are still higher than in developed countries. The median BLLs in Korean children aged 3–5 years was reported as 13.4 μg/L in 2014 [17]. In Japan, the geometric mean of BLLs in 12-year-old children was 7.0 μg/L [18]. In New Zealand and Canada, the geometric means of BLLs were reported as 8.6 μg/L [19] and 9.7 μg/L [20], respectively.

In the present study, we found that often hand washing before meals was inversely associated with BLLs, perhaps indicating that dust was a source of lead and that washing it off hands prevented exposure by inhalation or ingestion [21]. Therefore, children should wash their hands frequently, especially before eating, to reduce absorption of substances such as metals and bacteria. Furthermore, we found that BLLs exceeding 35 μg/L were more common in younger children (aged 1–3 years), however, there was no difference in the frequency of hand washing before meals between the younger (1–3 years old) and older (4–6 years old) children (data not shown). The reason for the inconsistent results could be that younger children have not yet developed good hygiene habits and they are more likely to put their contaminated hands in their mouths between meal times.

Food consumption is currently considered a major source of lead [7], but data on specific dietary sources of lead are inconsistent. We found that consumption of puffed grains was a risk factor for BLLs exceeding 20 μg/L, which was consistent with a previous report [22]. The main ingredients in puffed grain foods are starch, oil, and flavor additives, posing a further risk of overweight and obesity.

We also found an association between consumption of eggs and BLLs exceeding 20 μg/L, perhaps because environmental pollution increases lead content in eggs and lead absorption through the intestines increases with the number of eggs ingested. However, we did not find literature to support this view. Eggs contain a rich content of protein, and protein intake was reported to be a positive modulator of BLLs in humans [23]. Moreover, high-protein diets have been shown to increase lead absorption in rats [24]. Nonetheless, eggs are nutrient-rich foods that are easily accessible in low- and middle-income countries [25].

Previous studies have shown positive associations between consumption of grains and vegetables and BLLs, supporting the hypothesis that these foods are sources of lead [26–28]. Whole grains contain more dietary fiber than refined grains or vegetables, and dietary fiber can bind to lead and inhibit its gastrointestinal absorption [12, 29]. Other studies have reported that consumption of milk may also reduce lead uptake [30, 31], but we found associations with BLLs for only puffed grains and eggs, possibly reflecting geographic variations in dietary habits.

Our study had several limitations. First, this was a single-center study and some selection bias may be present. As children with lead poisoning is rare in China, blood lead testing is not routine inspection item. In our study, only healthy children whose parents asked for blood lead determination were investigated by questionnaire. Therefore, our selected population may have better health care awareness and have limitations in representing the general population in Shenyang. In addition, most data were collected by questionnaire and recall bias may be possible. Second, weighed food records provide quantitative information on individual diets and are considered a “gold standard” for dietary assessment. However, this method is time consuming and generally suitable only for individuals or small groups of cooperative volunteers [32]. Food frequency questionnaires are shown to be a practical and efficient approach to assess habitual diet over periods of time and are widely used as cost-effective dietary assessment methods in large-scale dietary surveys to investigate customary food intakes over extended periods of time [32]. Therefore, a food frequency questionnaire was used in the present study although the food frequency questionnaire may have some shortcomings. Third, the association between consumption of eggs and BLLs was significant only in the multivariate logistic regression model. Due to the small number of subjects and the fact that we cannot rule out the role of unmeasured confounders, the result should be interpreted with caution. Fourth, our study was cross-sectional and cannot infer a causal relationship.

In conclusion, we found that BLLs of children in Shenyang are still higher than in developed countries and that consumption of puffed grains and eggs is associated with higher BLLs in this cohort. Often hand washing before meals may be protective against high BLLs. Further prospective study is needed to verify these findings.

## Data Availability

The datasets generated during and analyzed during the current study are not publicly available due to privacy or ethical restrictions but are available from the corresponding author on reasonable request.

## Abbreviations

BLLs: Blood lead levels
GM: Geometric mean
GSD: Geometric standard deviation
CI: Confidence interval
OR: Odds ratio
